# Systematic review of the incidence and prevalence of genital warts

**DOI:** 10.1186/1471-2334-13-39

**Published:** 2013-01-25

**Authors:** Harshila Patel, Monika Wagner, Puneet Singhal, Smita Kothari

**Affiliations:** 1 LA-SER Analytics, 1405 TransCanada Highway, Suite 310, Montréal, Quebec H9P 2V9, Canada; 2 Merck & Co., Inc. Global Health Outcomes, Whitehouse Station, NJ, USA

**Keywords:** Anogenital warts, Condylomata acuminate, Epidemiology, Prevalence, Incidence

## Abstract

**Background:**

Anogenital warts (AGWs) are a common, highly infectious disease caused by the human papillomavirus (HPV), whose high recurrence rates contribute to direct medical costs, productivity loss and increased psychosocial impact. Because of the lack of a systematic review of the epidemiology of AGWs in the literature, this study reviewed the published medical literature on the incidence and prevalence of AGWs.

**Methods:**

A comprehensive literature search was performed on the worldwide incidence and prevalence of AGWs between 2001 and 2012 using the PubMed and EMBASE databases. An additional screening of abstracts from relevant sexual health and infectious disease conferences from 2009 to 2011 was also conducted. Only original studies with general adult populations (i.e., at least including ages 20 through 40 years) were included.

**Results:**

The overall (females and males combined) reported annual incidence of any AGWs (including new and recurrent) ranged from 160 to 289 per 100,000, with a median of 194.5 per 100,000. New AGW incidence rates among males ranged from 103 to 168 per 100,000, with a median of 137 per 100,000 and among females from 76 to 191 per 100,000, with a median of 120.5 per 100,000 per annum. The reported incidence of recurrent AGWs was as high as 110 per 100,000 among females and 163 per 100,000 among males. Incidence peaked before 24 years of age in females and between 25 and 29 years of age among males. The overall prevalence of AGWs based on retrospective administrative databases or medical chart reviews or prospectively collected physician reports ranged from 0.13% to 0.56%, whereas it ranged from 0.2% to 5.1% based on genital examinations.

**Conclusions:**

The literature suggests that AGWs are widespread and the prevalence depends on study methodology as suggested by higher rates reported from routine genital examinations versus those from treatment records. However, there remains a need for more population-based studies from certain regions including Africa, Latin America and Southern Asia to further elucidate the global epidemiology of this disease.

## Background

Anogenital human papillomavirus (HPV) is the most frequent sexually transmitted viral infection in the world, which can result in malignant cancers or benign skin and mucosal tumors, including anogenital warts (AGWs) [1]. AGWs are categorized as a clinical anogenital HPV infection because they manifest as visible lesions, namely as single or multiple papules on the vulva, perineum, perianal area, vagina, cervix, penis, anus, scrotum and urethra [1]. Clinical symptoms may include pruritus, burning, vaginal discharge and bleeding [2]. Four distinct sub-types of AGWs have been described: condylomata acuminata (pointed warts), flat / macular lesions, papular, and keratotic lesions [1]. The first two sub-types are mainly found on moist, non-keratinized epithelia, while the latter two usually present on keratinized epidermis [1]. AGWs are also often referred to as genital warts, condylomata acuminata or genital verruca, although strictly speaking the first two terms are subsets of the AGW category.

HPV 6 and 11 account for the majority of AGW cases [1,3-5]. AGWs are highly infectious; approximately 65% of individuals with an infected partner develop AGWs within 3 weeks and 8 months [6]. In rare cases, AGWs can be associated with malignant lesions, namely Buschke-Lowenstein tumors [5]. Recent prospective studies reported that the median time between infection with HPV types 6 or 11 and the development of AGWs was 11 to 12 months among males [7,8] and 5 to 6 months among young females [9]. Although there are no severe health implications or mortality associated with AGWs, there are significant psychosocial issues which often ensue [10,11].

Treatment options include patient-applied (home-based) chemical treatments (podofilox, imiquimod), physician-applied (office-based) chemical treatments (podophyllin, trichloracetic acid, interferon, green tea extract [12]) and ablative treatments (cryotherapy, surgical removal, laser treatment) [13-16]. The main limitation of current therapies is the high recurrence rate after initial remission [15,17,18]. The quadrivalent HPV vaccine demonstrated high efficacy in preventing the onset of HPV 6/11-related AGWs in both males [19] and females [20].

Although AGWs rank among the most frequent sexually transmitted diseases (STD) [21,22] the epidemiology of AGWs is not well characterized. A recent review by Scarbrough and colleagues reported the epidemiology of AGWs only in the USA, UK and France [23]. Syrjanen and colleagues evaluated the clinical burden of HPV 6 and 11 infections in Finland, including AGWs [24]. Other reviews summarized the epidemiology associated with HPV infections in general (including genital warts, oropharyngeal cancer and ano-genital cancers such as vulvar, vaginal, anal and penile cancers) [5,25,26]. Although providing important data, the primary focus of these reviews was not AGWs. Given the lack of systematic reviews focusing on the epidemiology of AGWs in the literature, the objective of this study was to review the recent published literature on the global epidemiology (incidence and prevalence) of AGWs in the general adult population.

## Methods

### Literature searches

The PubMed and EMBASE databases were searched for articles published from January 2001 through January 2012 in English, French, German, Spanish or Italian using the following search terms: (genital warts OR genital wart OR anogenital warts OR anogenital wart OR condyloma* OR genital verruca*) AND (prevalence OR incidence OR epidemiolog*). Database searches were followed by manual searches of bibliographies of selected references. Available abstracts of the following relevant sexual health and infectious disease conferences from 2009 to 2011 were also searched: International Society for Sexually Transmitted Diseases Research (2009–2011), International Papillomavirus Conference (2009–2011), Infectious Diseases Society of America (2010, 2011), European Research Organization of Genital Infection and Neoplasia (2009–2011), Centers for Disease Control and Prevention Advisory Committee on Immunization Practices (2009–2011), and International Conference on Emerging Infectious Diseases (2010, 2011).

Only original studies reporting AGW incidence, prevalence or self-reported history in the general adult (at least including ages 20 through 40 years) male, female or combined populations were selected. Studies focusing exclusively on children (age < 15 years) or adults within a narrow age range, those with study populations composed of immunocompromised individuals (e.g., HIV-infected) only, high-risk populations (e.g., men who have sex with men, sex workers), single-center studies (not reporting on epidemiology in the general population), case reports, commentaries, narratives or reviews were excluded.

### Data extraction and analysis

Data extracted from all articles included the year of study, country (and sub-region, if relevant), setting, population, age groups, methodology, annual incidence (categorized into new, recurrent, and any cases, if available) overall and by age subgroup, prevalence (%) overall and by age subgroup, self-reported history of AGWs (within the past 12 months or lifetime) and temporal trends in the incidence of AGWs for males, females and overall. Reported incidence rates were recalculated per 100,000 population, if originally not reported as such. AGWs reported as ‘new’ or ‘incident’ were both termed ‘new’ in this review. For incidence and prevalence data collection, the methods consisted of extracting data from articles that included: (1) retrospective administrative databases or medical chart reviews, (2) prospectively collected physician reports, and (3) genital examinations performed by a healthcare provider on individuals from the general population visiting clinics. The age groups experiencing the highest incidence of AGWs and the incidence at that peak age were also recorded for each study, where available. Data across different sources were synthesized using descriptive statistics, including medians and ranges, where appropriate. For studies that only reported incidence for males and females separately, the average was taken to represent the overall incidence. The searches and data extraction were conducted by two investigators using the same methodology. In cases of disagreements, results were reconciled through mutual discussion.

## Results

**Figure 1:**
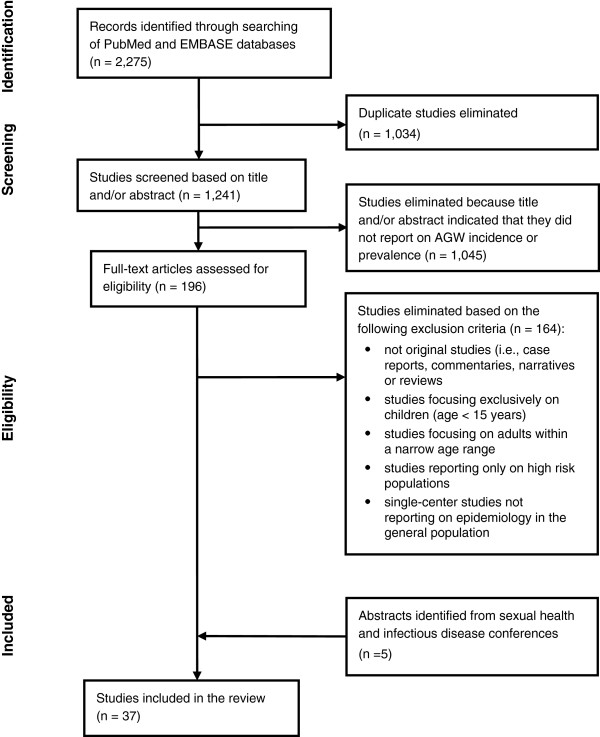
Literature review.

PubMed and EMBASE searches for studies on the epidemiology of AGWs yielded 802 and 1,473 records, respectively, for a total of 2,275 records (Figure 1). After elimination of duplicates, 1,241 records were screened and 1,045 were excluded because the title and/or abstract indicated that they did not report on AGW incidence or prevalence. The remaining 196 studies were examined in full-text for eligibility for this review. Of these, 32 studies met the inclusion criteria: 12 from Europe, 10 from North America (including Mexico), 4 from Asia, 3 from South America, 2 from Australia and 1 multiregional. Screening abstracts from relevant conferences yielded an additional 5 references: 3 from Europe and 1 each from Canada and Japan (Figure 1).

Studies included in the analysis are summarized in Tables 1 and 2, on incidence and prevalence, respectively. Each table is subdivided into three categories based on methodology of data collection. Table 3 summarizes data from surveys that asked members of the general population about their history of clinically diagnosed AGWs.

**Table 1:** Incidence of anogenital warts

**Study**	**Country / Year**	**Setting, population and age groups**	**Annual incidence per 100,000**			**Definitions**
			**New**	**Recurrent**	**Any (including new and recurrent)**	
**Data based on retrospective administrative databases or medical chart reviews**						
Pirotta et al., 2010 [27]	Australia / 2000-06	**Setting**: nationally representative random sample of approximately 6,460 practicing GPs and sexual health clinics	NA	NA	Overall: 219 Males: 206 Females: 231	Any: GW cases never managed by any doctor or a first medical consultation for a new episode of a recurrent problem as determined by GP at consultation
		**Population:** general population				
		**Age**: all ages				
Steben et al., 2011 [31]	Canada (Quebec) / 2007 (data for most recent year)	**Setting**: provincial, public healthcare system	Males: 103 Females: 98	NA	NA	New: GW cases preceded by a 12-month, disease-free period of GW care
		**Population**: population covered by the public drug plan (41% of total population)				
		**Age**: all ages				
Marra et al., 2009 [32]	Canada (British Columbia) / 1998-2006	**Setting**: physicians, hospitals and STI clinics (province-wide)	Overall: 126 Males: 131 Females: 121	NA	NA	New: GW cases preceded by a 12-month, disease-free period of GW care
		**Denominator**: general population				
		**Age**: all ages				
Kliewer et al., 2009 [33]	Canada (Manitoba) / 2004	**Setting**: physician offices and hospitals (province-wide)	Males: 154 Females: 120	NA	NA	New: GW cases preceded by a 12-month, disease-free period of GW care
		**Population**: general population				
		**Age**: all ages				
Kraut et al., 2010 [34]	Germany / 2005-06	**Setting**: physician offices and hospitals	Overall: 170 Males: 148 Females: 191	NA	NA	New: GW cases preceded by a 12-month, disease-free period of GW care
		**Population**: a sample of the German population with statutory health insurance covering all geographical regions (N = 14 million)^†^				
		**Age**: 10–79 years				
Vittori et al., 2008 [28]	Italy / 2005	**Setting**: random sample of 78 gynecologists stratified by geographic region	Females: 430	Females: 110 (early); 60 (late)	NA	New: patients without previous history of GWs. Early recurrent: patients who had a GW episode within the previous 12 months
		**Population**: female population visiting gynecologists				Late recurrent: patients who had a GW episode more than 12 months prior to study episode
		**Age**: 14–64 years				
van den Broek et al., 2010 [39]	Netherlands / 2006	**Setting**: GPs (representative national sample) and all Dutch STI centers	NA	NA	Males: 94* Females: 137*	Any: patients with symptoms of GWs
		**Population**: general population				
		**Age**: all ages				
Castellsague et al., 2009 [29]	Spain / 2005	**Setting**: representative sample of dermatologists, gynecologists and urologists in the public healthcare setting from 6 autonomous regions	Overall: 118 Males: 137 Females: 100	Overall: 43 Males: 47 Females: 39	Overall: 160 Males: 184 Females: 137	New: patients without any prior GW diagnosis
		**Population**: general population (68% of total Spanish population)				Recurrent: patients who had a previous GW episode
		**Age**: 14–64 years				
Cassell et al., 2006 [40]	UK / 1998-2000	**Setting**: representative sample of GPs and GUM clinics	NA	NA	Males: 308 Females: 236	Any: all patients diagnosed with GWs
		**Population**: general population				
		**Age**: all ages				
Desai et al., 2011 [38]	UK (England) / 2006-2009	**Setting**: representative sample of GPs and all GUM clinics	Overall: 157 Males: 168 Females: 142	Overall: 133 Males: 163 Females: 103	Overall: 289 Males: 331 Females: 245	New: patients diagnosed with GWs for the first time during study period
		**Population**: general population				Recurrent: episode occurring more than 8 weeks after last attendance
		**Age**: all ages				
Hoy et al., 2009 [35]	USA / 2004	**Setting**: physicians, hospitals, ERs reimbursed by 5 geographically dispersed private health plans	Overall: 120 Males: 110 Females: 120	NA	NA	New: individuals not having a medical or pharmacy claim associated with GWs in the previous 12 months to the index medical claim
		**Population**: privately-insured population (N = 3.8 million)				
		**Age**: all ages				
Insinga et al., 2003 [2]	USA / 2000	**Setting**: physicians, hospitals, ERs reimbursed by private health plans	NA	NA	Overall: 170 Males: 167 Females: 165	Any: all patients diagnosed with GWs
		**Population**: privately-insured population (N = 3.7 million)				
		**Age**: all ages				
Koshiol et al., 2004 [36]	USA / 2001	**Setting**: physicians, hospitals, ERs reimbursed by approximately 30 private health plans (national)	Overall: 205	NA	NA	New: no GW-related claims during the initial 12 months of continuous enrollment
		**Population**: privately-insured population (N = 5.9 million)				
		**Age**: 15–59 years				
**Data based on prospectively collected physician reports**						
Lin et al., 2010 [30]	China (Hong Kong) / 2009	**Setting**: sample of 170 physicians operating in private clinics (GPs, obstetricians/gynecologists and dermatologists/venereologists) and all 5 local social hygiene clinics	Overall: 204 Males: 292 Females: 125	NA	NA	New: cases with no past symptoms or clinical diagnosis of GWs
		**Population**: general population				
		**Age**: ≥18 yrs				
Monsonego et al., 2007 [43]	France / 2005	**Setting**: nationwide random sample of 212 gynecologists	Females: 176	Females: 48	Females: 229	NA
		**Population**: general female population				
		**Age**: 15–65 years				
Hillemanns et al., 2008 [37]	Germany / 2005	**Setting**: representative sample of 129 gynecologists	Females: 114 (age 14–65); 76 (all ages)	Females: 35 (age 14–65); 23 (all ages)	Females: 149 (age 14–65); 99 (all ages)	New: patients diagnosed with GWs at the time of the visit
		**Population**: general female population				Recurrent: patients with previous episodes of GWs that had resolved
		**Age**: 14–65 years and all ages				Resistant: patients with previous episodes of GWs that had not resolved with treatment
Pasciullo et al., 2011 [41]	Italy / 2009	**Setting**: geographically representative sample of 650 GPs	NA	NA	Overall: 8 Males: 10 Females: 4	Any: all patients diagnosed with GWs
		**Population**: population listed with the above GPs (N = 959,778)				
		**Age:** ≥15 years				
**Data based on genital examinations of samples from the general population**						
Anic et al., 2011 [8]	Multinational (USA, Mexico, Brazil) / 2005-2009	**Setting**: prospective study investigating the natural history of HPV infection in men (HPV in Men study)	Males: 235	NA	NA	NA
		**Population**: males with no previous history of GW participating in the above study (N = 2,487)				
		**Age**: 18–70 years				
Sasagawa et al., 2011 [42]	Japan / 2009	**Setting**: 63 private clinics in 5 districts	NA	NA	Females: 251	NA
		**Population**: women attending Pap screening in above private clinics (N = 60,414)				
		**Age**: 10–59 years				

*AGWs *anogenital warts, *ER *emergency room, *GP *general practitioner, *GUM *genitourinary medicine, *GWs *genital warts, *N* number of individuals examined or followed, *NA *not available, *STI* sexually transmitted infection.

* GP and STI center rates combined.

†About 90% of the German population is covered by the statutory health insurance [34].

**Table 2:** Prevalence of anogenital warts

**Study**	**Country / year**	**Population, setting and age groups**	**Prevalence %**		
			**Males**	**Females**	**Overall**
**Data based on retrospective administrative databases or medical chart reviews**					
Marra et al., 2009 [32]	Canada (British Columbia) / 2006	**Setting**: physicians, hospitals and STI clinics (province-wide)	0.16	0.14	0.15
		**Denominator**: general population			
		**Age**: all ages			
Kliewer et al., 2009 [33]	Canada (Manitoba) / 2004	**Setting**: physician offices and hospitals (province-wide)	0.17	0.13	0.15
		**Population**: general population			
		**Age**: all ages			
Vittori et al., 2008 [28]	Italy / 2005	**Setting**: random sample of 78 gynecologists stratified by geographic region	NA	0.6	NA
		**Population**: female population visiting gynecologists			
		**Age**: 14–64 years			
Castellsague et al., 2009 [29]	Spain / 2005	**Setting**: representative sample of dermatologists, gynecologists and urologists in the public healthcare setting from 6 autonomous regions	0.20	0.16	0.18
		**Population**: general population (68% of total Spanish population)			
		**Age**: 14–64 years			
**Data based on prospectively collected physician reports**					
Mariani et al., 2011 [47]	Italy / 2010	**Setting**: 45 extra-hospital gynecologists in gynecological ambulatories stratified by geographic region	NA	0.56	NA
		**Population**: female population listed with the above GPs (N = 16,410)			
		**Age**: 15–64 years			
Pasciullo et al., 2011 [41]	Italy / 2009	**Setting**: geographically representative sample of 650 GPs	0.06	0.03	0.05
		**Population**: population listed with the above GPs (N = 959,778)			
		**Age**: ≥15 years			
Lee et al., 2010 [46]	South Korea / 2008	**Setting**: gynecological clinics in the top 6 metropolitan cities	NA	0.14	NA
		**Population**: female patients visiting the above gynecological clinics (N = 117,381)			
		**Age**: all ages			
**Data based on genital examinations of samples from the general population**					
Sellors et al., 2000 [48]	Canada (Ontario) / 1998-1999	**Setting**: family practices for cytologic screening randomly selected in proportion to the regional population	NA	1.10	NA
		**Population**: female population attending cervical Pap screening with the above family physicians (N = 909)			
		**Age**: 15–49 years			
Nyári et al., 2004 [49]	Hungary (Southeast) / 2000	**Setting**: outpatient gynecology clinics	NA	4.03	NA
		**Population**: randomly selected asymptomatic female population attending the above gynecology clinics (N = 397)			
		**Age**: mean 35.5 years (SD 9.7)			
Jimenez-Vieyra, 2010 [50]	Mexico (Mexico City) / 2002–09	**Setting**: sexual health clinic	NA	3.20	NA
		**Population**: female population attending opportunistic cervical Pap screening at above sexual health clinic (N = 3,232*)			
		**Age**: 15–54 years			
Vaccarella et al., 2006 [45]	Mexico / 2003-2004	**Setting**: 27 public clinics stratified by geographic region	5.1^†^	NA	NA
		**Population**: male population seeking vasectomy in the above clinics (N = 779)			
		**Age**: mean 34.0 years			
Garcia et al., 2004 [51]	Peru / 1997-98	**Setting**: CBOs (mother’s clubs) from 18 districts	NA	2.40	NA
		**Population**: rural female population from the above mother’s clubs (N = 752)			
		**Age**: 18–67 years			
Nyitray et al., 2008 [44]	USA (Tucson and Tampa) / NA	**Setting**: prospective HPV epidemiology study	4.10	NA	NA
		**Population**: heterosexual men from general population participating in the above study (N = 222)			
		**Age**: 18–40 years			
Lan et al., 2008 [52]	Vietnam (Bavi district) / 2006	**Setting**: gynecological examinations performed by physicians	NA	0.20	NA
		**Population**: married rural women randomly recruited from the general population (N = 1,012)			
		**Age**: 18–49 years			

*AGWs *anogenital warts, *GP *general practitioner, *GWs *genital warts, *N *number of individuals examined or followed, *NA *not available, *SD *standard deviation, *STI *sexually transmitted infection.

* Number of Pap tests performed.

^† ^Penile condyloma acuminata.

**Table 3:** Past 12-month and lifetime history of anogenital warts based on self reports

**Study**	**Country / year**	**Population, setting and age groups**	**Self-reported history of AGWs, % (95% CI)**	
			**Within the past 12 months**	**Lifetime**
Matos et al., 2003 [58]	Argentina / 1998	Random sub-sample of females from 1,786 households stratified by socio-economic status; age: ≥ 15 years; N = 1,028	NA	1.8
Syrjanen et al., 2005 [59]	Argentina and Brazil / 2002–2003	Women attending four clinics as part of the Latin American Screening Study multi-center screening trial; age: 14–67 years; N = 12,107 (Argentina, [Buenos Aires]: N = 3,437; Brazil [Campinas]: N = 2,627; [Porto Alegre]: N = 3,043; [Sao Paolo]: N = 3,000)	NA	Vulvar / anal warts:
				Buenos Aires: 0.4 / 0
				Campinas: 2.3 / 0.3
				Porto Alegre: 3.4 / 0.5
				Sao Paolo: 1.1 / 0.1
Brotherton et al., 2009 [55]	Australia / 2001-02	Representative sample of the general population; age: 16–59 years; N = 9,729 males and 9,578 females	Males: 0.5*	Males: 4.0*
			Females: 0.3*	Females: 4.4*
Parish et al., 2007 [61]	China	Representative sample of the general population; age: 20–64 years; N = 2,999	Males: 1.2 (0.7–2.0)	NA
			Females: 2.0 (1.2–3.2)	
Blomberg et al., 2010 [60]	Denmark	Random sub-samples of males and females from the general population; age: 18–45 years; N = 23,080 males	NA	Males: 7.9*
Kjaer et al., 2007 [54]	Denmark, Iceland, Norway and Sweden / 2004-05	National probability sample of general female population; age:18–45 years; N = 22,199 Denmark; 15,051 Iceland; 16,604 Norway; 15,713 Sweden	Denmark: 1.3 (1.2–1.5)*	Denmark: 10.1 (9.7–10.5)*
			Iceland: 1.9 (1.7–2.1)*	Iceland: 12.0 (11.5–12.6)*
			Norway: 1.1 (1.0–1.3)*	Norway: 9.5 (9.0–9.9)*
			Sweden: 1.0 (0.9–1.2)*	Sweden: 11.3 (10.8–11.8)*
Fenton et al., 2001 [56]	England, Scotland and Wales / 1999-2001	National probability sample of sexually active population; age: 16–44 years; N = 5,376 males and 5,323 females	NA	Males: 3.6 (3.1–4.2)*
				Females: 4.1 (3.6–4.7)*
Klavs and Grgic-Vitek, 2008 [53]	Slovenia / 1999-2001	National probability sample of sexually active population; age: 18–49 years; N = 752 males and 842 females	NA	Males: 0.27 (0–1.3)*
				Females: 0.36 (0.1–1.1)*
Dinh et al., 2008 [57]	USA / 1999-2004	National probability sample of sexually active general US population; age: 18–59 years; N = 4,673 females and 4,176 males	NA	Males: 4.0 (3.2–5.0)*
				Females: 7.2 (6.2–8.4)*

* In these surveys, participants were specifically asked about clinically diagnosed warts.

*CI *confidence interval, *N*: number of individuals examined or followed, *NA *not available.

### Incidence of anogenital warts

Thirteen studies reported the incidence of new cases of AGWs (Table 1), defined as diagnosed for the first time at a consultation with no past symptoms or clinical diagnosis of AGWs [27-30] or cases with no AGW-related claim in the previous 12 months [31-36]. Five studies reported the incidence of recurrent AGWs (Table 1), whose definition varied. It included a previous AGW episode that had resolved [29,37], an episode occurring more than eight weeks after the last caregiver attendance [38], or an episode occurring within 12 months of a previous AGW episode (early recurrence) or more than 12 months after a previous episode (late recurrence) [28]. Ten studies reported the incidence of any AGWs (Table 1), frequently defined as new and recurrent combined.

In the overall population (males and females combined) the annual incidence of any AGWs (including new and recurrent) ranged from 160 in Spain [29] to 289 in the United Kingdom [38] per 100,000, with a median of 194.5 per 100,000 across four studies (Table 1) [2,27,29,38]. The overall annual incidence of new cases ranged from 118 in Spain to 205 in the US per 100,000, with a median of 157 per 100,000 across seven studies [29,30,32,34-36,38].

The regional distribution of new cases of AGWs per 100,000 population was as follows: 101 to 205 in North America [31-33,35,36], 118 to 170 in Europe [29,34,38] and 204 in Asia (Table 1) [30].

Among males, the overall annual incidence of any AGWs ranged from 94 in the Netherlands [39] to 331 in England [38] per 100,000 general population, with a median of 195 [2,27,29,38-40]. An Italian study [41] reported an incidence outside this range of 10 GW cases diagnosed by GPs per 100,000 males. The reported incidence of new AGWs per 100,000 males per year ranged from 103 among the male population of Quebec, Canada, that is covered by the public drug plan [31] to 168 in England [38], with a median of 137, across seven studies in Europe and North America that were based on administrative records or chart reviews (Table 1) [29,31-35,38]. One prospective study from Hong Kong [30] reported a rate outside this range, at 292 new cases per 100,000 men per year, and another, the multinational prospective HPV in Men study that followed 2,487 men from Florida (USA), Sao Paulo (Brazil) and Morelos (Mexico) with repeated genital examinations reported a new AGW incidence of 235 cases per 100,000 person-years [8] (Table 1). Two studies estimated the rate of recurrent AGWs in males [29,38] (Table 1). One of them (from the UK [38]), defining recurrent AGWs as those with a new medical consultation occurring at least eight weeks after the last AGW-related consultation, reported a rate (163 per 100,000) that was almost as high as that of new cases. The other study (from Spain [29]), defining recurrent cases as “those that had a previous episode”, reported a much lower rate of 47 per 100,000 men.

Among studies with the general female population, the reported annual incidence of any AGWs (including new and recurrent) per 100,000 population ranged between 99 among women of all ages in Germany [37] and 251 among 10- to 59-year-old women attending Pap screening in Japan [42], with a median of 224 (Table 1) [2,27,29,37-40,42,43]. The Italian prospective study [41] reported a much lower incidence compared to this range, of four cases per 100,000 females listed with GPs. Annual incidence of new AGWs per 100,000 females ranged between 76 among women of all ages in Germany accounting only for AGWs diagnosed by gynecologists [37] and 191 in another German study taking into account AGWs diagnosed in women aged 10 to 79 years in physician offices and hospitals [34], with a median of 120.5 (Table 1) [29-35,37,38,43]. An Italian study reported an annual incidence of 430 new AGW cases per 100,000 females seen by gynecologists [28]. Incidence of recurrent AGWs ranged between 23 in Germany [37] and 103 in England [38] cases per 100,000 females per year (Table 1) [29,37,38,43].

**Figure 2:**
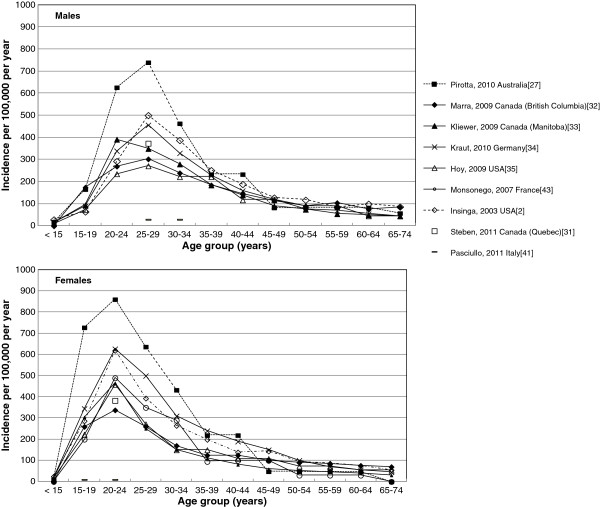
Age-specific incidence of anogenital warts in males and females.

Among the studies reported above, eight provided data on age-specific AGW incidence for both males and females separately and one for females only (Figure 2) [2,27,31-35,41,43]. Among males, incidence peaked in the 25- to 29-year age group in six studies [2,27,31,32,34,35] and in the 20- to 24-year age group in one study [33], anging between 272 (new cases in the USA [35]) and 740 (new and recurrent cases in Australia [27]) per 100,000 (Figure 2) [2,27,31-35,38]. Among females, incidence generally peaked in the 20-year age groups ranging from 338 (new cases in British Columbia, Canada [32]) to 861 (new and recurrent cases in Australia [27]) per 100,000 (Figure 2) [2,27,31-35,43]. One study from England, reported a peak incidence of 755 new cases per 100,000 individuals aged 20 to 24 years (males and females combined; not shown in Figure 2) [38]. The Italian prospective study (Pasciullo, 2011 [41]) reported peak incidence rates outside these ranges: 30 per 100,000 males aged 25 to 34 years and 10 per 100,000 females aged 15 to 24 years. In six of the eight studies that reported data for both genders [2,27,31-35,41], AGW incidence peaked in a younger age group among females than among males. AGW incidence remained significant in the 30- to 45-year age group, ranging from approximately 110 to 290 per 100,000 among females [2,27,32-35,43] and from 190 to 310 cases per 100,000 among males [2,27,32-35].

### Prevalence

Overall, AGW prevalence ranged from 0.15% to 0.18% based on administrative databases or chart reviews that used the general population as the denominator (Table 2) [29,32,33].

Among males, prevalence ranged from 0.16% to 0.20% (Table 2) [29,32,33]. Prevalence estimates were higher among two studies that were based on genital examination of males from general population samples: 4.1% among heterosexual, sexually active US men (age 18–40 years) who agreed to participate in a prospective epidemiological study and denied a previous history of AGWs [44] and 5.1% (penile condyloma) among males seeking a vasectomy in public clinics in Mexico [45] (Table 2). A lower prevalence (0.06%) was estimated in the Italian prospective study [41] based on males listed with a geographically representative sample of GPs (Table 2).

Among females, prevalence ranged between 0.13% and 0.16% in studies using the general female population as the denominator [29,32,33] and ranged between 0.03% and 0.6% in four studies using the population consulting gynecologists as a denominator (Table 2) [28,41,46,47]. In studies based on gynecologic examination of women from the general population or those attending cervical Pap screening [48-52], reported prevalence ranged between 1.1% in Canada [48] and 4.0% in Hungary [49], with the exception of married rural women in Vietnam (0.2%) [52] (Table 2).

### Self-reported history of genital warts

In surveys of general adult populations (Table 3), 0.36% (Slovenia, sexually-active, age 18–49 years [53]) to 12.0% (Iceland, age 18–45 years [54]) of females reported a lifetime history of genital warts [53-59]. The corresponding proportion in the male population varied from 3.6% to 7.9% in Australia, Denmark, the UK and the USA and was 0.27% in Slovenia (Table 3) [53,55-57,60]. An AGW history in the last 12 months was reported by 0.3% of females in Australia [55], 1.0% to 1.9% of females in the Nordic countries [54] and 1.2% of males and 2.0% of females in China [61] (Table 3).

### Comparison between males and females

Among thirteen studies based on retrospective administrative databases or medical chart reviews, prospectively collected physician reports or genital examinations providing incidence or prevalence estimates for both sexes, nine reported higher rates for males than for females (Tables 1 and 2) [2,29-33,38,40,41]. However, in surveys that included both genders, more females than males admitted ever having had AGWs (Table 3) [53,55-57,61].

### Temporal trends

**Figure 3:**
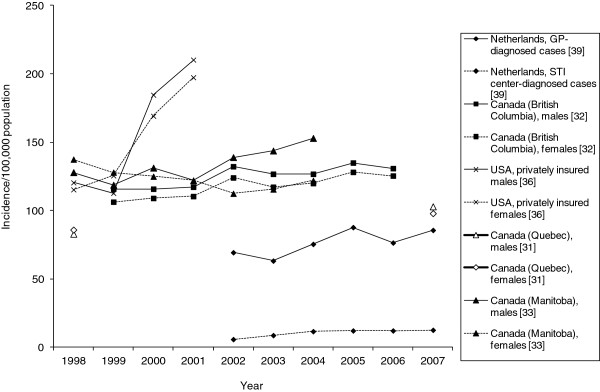
**Temporal trends in the incidence of anogenital warts. **GP: general practitioner; STI: sexually transmitted infection.

Five studies [31,32,36,39,54] provided data with respect to temporal trends of AGW epidemiology shown in Figure 3. A population-based study from British Columbia, Canada, reported that the annual AGW incidence increased significantly from 107 per 100,000 in 1999 to 126 in 2006 (*P* < .0001); increases were observed among both males and females [32]. Prevalence also increased during this time period from 0.11% to 0.15% (*P* < .001). Another study of individuals covered by the public drug plan in Quebec, Canada reported that the annual AGW incidence increased from 83 to 103 per 100,000 among males from 1998 to 2007 whereas among females, it increased from 86 to 98 per 100,000 from 1998 to 2002 [31]. A US study evaluating health claims from privately insured individuals reported an increase in AGW claims from 118 (95% CI 110–126) to 205 (95% CI 199–211) per 100,000 person-years at risk from 1998 to 2001, representing an increase of greater than 70% [36]. A study in the Netherlands observed a significant linear increase in AGW diagnoses from 2002 to 2007 (no *P* value provided), particularly those diagnosed by general practitioners [39]. Using self-reported data from a random sample of the general female population in Denmark, Sweden, Norway and Iceland born between 1958 and 1986, Kjaer and colleagues [54] found that the age-specific cumulative incidence, estimated based on age at first diagnosis, increased with each subsequent younger birth cohort (*P* < .01), an effect that was most pronounced in Iceland and Norway (not shown in figure).

## Discussion

AGWs are a common manifestation of an HPV infection, particularly among young men and women. Reported annual incidence rates typically range between 100 and 200 new cases per 100,000 general adult population based on retrospective administrative databases, medical chart reviews and prospectively collected physician reports. AGW prevalence estimates typically range between 0.13% and 0.20% among these studies. There were also no marked regional differences observed in the incidence and prevalence of AGWs. These methodological approaches cover large populations; however, they can only capture AGW cases among patients seeking care in their respective healthcare systems. Studies which are based on genital examination of general population samples are less dependent on healthcare seeking behavior and tend to report comparatively higher prevalence estimates (ranging from 1% to 5%). However, prevalence data for males based on genital examinations of asymptomatic individuals are quite limited because, unlike females who routinely visit their gynecologists, males usually seek consultation with a reproductive specialist (i.e. urologists) if they present symptoms. Given these data limitations, our review includes a Mexican study of males seeking vasectomies in public clinics [45] and a prospective HPV epidemiology study of heterosexual males [8], populations, although not strictly general, may approximate the general male population.

The specialty of the physicians most frequently performing the initial diagnosis of AGWs varies depending on the healthcare system of individual countries, which could contribute to the differences in reported AGW incidence rates across studies. According to national database studies in both the US [36] and Germany [34], most females visited their gynecologists, while males consulted primarily dermatologists. In the United Kingdom, both males and females were most frequently diagnosed in genitourinary clinics [38,40]. In the Netherlands, individuals with AGWs are usually diagnosed by general practitioners [39].

Patients who contract AGWs may have a limited capacity to recognize them or be unwilling or unable to seek treatment. This may be due to a variety of psychological and social reasons, both of which could explain why prevalence estimates tend to be higher among studies based on genital examination. The Multicenter AIDS Cohort Study, including men both with and without HIV infection, found that, of men with external AGWs diagnosed by a trained clinician, only 38% reported having genital warts [62]. Similarly, one study among heterosexual men who denied a history of genital warts reported that on clinical examination, 4.1% of them had visible warts on genital sites [44]. With respect to healthcare seeking behavior, one survey among males and females with AGWs across Canada, France, Germany, the UK, and the USA observed that one third of respondents delayed seeking healthcare hoping that the warts would clear without treatment or thinking that the problem was not serious [11]. Spontaneous remission of AGWs is possible, but the reported proportions of individuals who actually experience spontaneous clearance vary widely from 0% to 50% [13,18]. Furthermore, there is a lack of a published systematic analysis of this issue. A Canadian study reported that the median delay between the time patients first noticed they had AGWs and their first visit to a healthcare provider was 76 days for men and 30 days for women [63]. Obviously, all individuals participating in these studies eventually contacted the healthcare system for their condition [11]. Thus, the true number of individuals being aware of having AGWs but never seeking healthcare would be difficult to estimate. In this context it is interesting to note that in studies based on medical or administrative records, AGW incidence rates tend to be higher among males than females; however, in population-based surveys, consistently more females than males disclose a history of AGWs [53,55-57].

Potential changes in treatment-seeking behavior could explain recent increases in AGW incidence, observed by several European and North American studies [31,32,36,39,54]. However, to our knowledge, there is little evidence that such changes occurred in the time periods analyzed. According to one study, younger women in Denmark, Sweden, Norway and Iceland tended to report higher numbers of sex partners than older birth cohorts, and lifetime number of sex-partners was a strong correlate of self-reported AGW history [54]. Thus, changes in sexual behavior may potentially contribute to increased AGW diagnoses reported in some studies.

The epidemiological data consistently confirms that AGW incidence peaks in young males and females, corresponding to the age of peak rate of new partner acquisition [64]. The earlier peak among females than males could be related to sexual mixing patterns, as younger females tend to have older male sex partners [64,65], or, possibly, to shorter incubation times in females [7-9], although directly comparative data is currently not available. A UK study reported that among AGW patients, the loss of quality of life was greatest for women in the youngest age group (16-19 years) [66].

The annual incidence of recurrent AGWs ranged between 47 and 163 cases per 100,000 males and between 23 and 110 per 100,000 females in population-based studies [28,29,37,38,43]. Although some of the variation may be due to differences in case definitions, particularly with respect to the length of symptom-free interval for an episode to be counted as recurrent, this finding nevertheless highlights the high burden of recurrent disease. Recurrence rates observed in clinical trials of AGW therapies range widely among studies and treatments, from 9% to 80% [13,14,16,18]. One retrospective analysis including 289 patients attending an STD clinic in Copenhagen, Denmark, reported that 65% of the patients had at least one recurrent AGW episode [67].

The high incidence of AGWs and the substantial economic and psychosocial burden of this condition and its treatment [68] indicate that it would be more beneficial to prevent rather than treat AGWs. The Centers for Disease Control and Prevention (CDC) recommend several options to reduce the risk of contracting AGWs including the correct and consistent use of condoms and altering sexual behavior by limiting the number of sexual partners [69]. The CDC also recommends the currently licensed quadrivalent HPV vaccine [69], which has shown high prophylactic efficacy against HPV 6/11-related genital warts in females [70] and males [19].

There are limitations to our study. One of them is our focus on peer-reviewed literature published in the last 10 years. This limitation was a consequence of the large scope of this review that intended to comprehensively capture the epidemiology of the disease, while being global in nature and rigorous in data extraction and analysis. Other limitations stem from gaps in the literature. Despite the substantial literature available on the epidemiology of AGWs, they are a notifiable disease in the UK only, which provides extensive incidence data [10]. However, there is limited epidemiological data for certain European countries, particularly Eastern Europe and little data from other regions of the world, such as Africa, Latin America and Southern Asia.

Further, some incidence rates have to be interpreted with caution because of variations in the methodologies and age ranges of study populations across the studies included. Some studies included AGW patients of all ages, whereas others only evaluated an age range that represented a more sexually active population (e.g., age 14 to 64). The latter can overestimate the reported incidence rate as is demonstrated by the data of Hillemanns and colleagues [37]. In this study, the incidence of any AGWs was 149 per 100,000 women aged 14 to 65 years compared to 99 for the entire female population [37]. Furthermore, Kraut and colleagues noted that in Germany the incidence was highest in the city states of Hamburg, Bremen and Berlin compared to other, less urbanized German regions [34]. If the same were true for Asia, the incidence reported from Hong Kong [30] and from the six largest metropolitan centers in South Korea may not be applicable country-wide [46]. An Italian study [41] reported AGW incidence rates in males and females that where substantially below the ranges reported by other studies, possibly because it only captured AGWs diagnosed by GPs excluding AGWs diagnosed by specialists (e.g., gynecologists, dermatologists). Similarly, a Quebec study reporting AGW incidence among individuals covered by the public drug plan may overrepresent the elderly population and underestimate this rate among the younger, working population, which is usually covered by private drug plans [31]. Nevertheless, this review provides a comprehensive description of the epidemiology of AGWs based on the available published literature.

## Conclusion

AGWs are common in both males and females across the world but data is limited, partially because, in contrast to some other STDs, their reporting is not mandatory. Further population-based studies are required to arrive at a more accurate representation of the global epidemiology of AGWs, to help policy makers make informative decisions about adopting effective treatment and preventative practices.

## Competing interests

This study was funded by Merck & Co., Inc. Harshila Patel PhD and Monika Wagner PhD are employees of LA-SER Analytics, who were paid consultants to Merck & Co., Inc. in connection with the development of this manuscript. Puneet Singhal PhD and Smita Kothari PhD are employees of Merck & Co., Inc. The authors declare that they have no non-financial competing interests.

## Authors’ contributions

HP and MW carried out the systematic review of the literature and drafted the manuscript. All authors were involved in the conception of the study. All authors read and approved the final manuscript.

## Pre-publication history

The pre-publication history for this paper can be accessed here:

http://www.biomedcentral.com/1471-2334/13/39/prepub
